# Microsurgical reconstruction after foot eumycetoma excision: Description of two cases

**DOI:** 10.1016/j.jpra.2025.11.019

**Published:** 2025-12-11

**Authors:** Matilde Mariani, Martin Lhuaire, Etienne Canoui, Patrick Knipper, Ignacio Garrido, David Biau, Laurent A. Lantieri, Victor Pozzo

**Affiliations:** a Service de Chirurgie Plastique, Hôpital Européen Georges Pompidou, Assistance Publique-Hôpitaux de Paris (APHP), Université Paris Descartes, Paris, France; b In-vivo Imaging Research Lab, Paris Cardiovascular Research Center – Inserm U970, Hôpital Européen Georges Pompidou, Université Paris Cité, Paris, France; c Department of Anatomy and Organogenesis, School of Medicine, Université Paris Cité, Paris, France; d Service de Chirurgie Orthopédique, Hôpital Cochin, Paris, France

**Keywords:** Microsurgery, Lower limb reconstruction, Infectious

## Introduction

Eumycetoma is a chronic granulomatous inflammatory disease caused by fungi, primarily affecting lower limbs, especially the foot. It is characterized by slowly progressing painless nodules and discharging sinus tracts. Advanced disease can invade deeper structures such as fascia, tendons, and bone.^1^, ^2^, ^3^ Although most prevalent in tropical and subtropical regions–the so-called “mycetoma belt”–with reported village-level prevalence as high as 1–5 cases per 1000 inhabitants in Sudan, cases are increasingly reported in non-endemic areas, including Europe, primarily in immigrants from endemic countries.^2^^,^^4^^,^^5^ Management requires a multidisciplinary approach, with long term systemic antifungals along with surgical debulking playing a central role due to the limited penetration of antifungal agents into the fibrotic lesion. The surgical approach often necessitates wide excision to minimize recurrence, which remains high—up to 50 % in some series. In advanced cases extensive surgery can be debilitating and may lead to amputation if reconstruction is not feasible.^3^^,^^6^^,^^7^

Microsurgical free flaps allow for immediate soft tissue coverage after the most possible radical resection, preserving limb function and aesthetics while promoting faster healing. Despite their potential in eumycetoma treatment, free flaps are rarely reported in literature. This article reports two non-endemic cases treated with wide en-bloc excision and immediate free flap reconstruction, highlighting the feasibility and advantages of microsurgical reconstruction in select patients.

## Patients and methods

This report describes two male patients treated with wide en-bloc excision and immediate microsurgical free flap reconstruction for recurrent eumycetoma of the foot. Surgeries were performed at a tertiary referral center by orthopedic oncology and plastic surgery teams.

### Case 1

#### Patient characteristics

A 44-year-old Mauritanian man, living in France since 2009, presented in May 2023 with recurrent eumycetoma on the dorsum of the right forefoot. He had hypertension, dyslipidemia, and a BMI of 23.5. Symptoms began in 2005, but he delayed medical attention for 10 years. Primary surgeries were performed in 2015 and 2019.

#### Presentation and imaging

The 4 × 3 cm mass was polylobulated, firm, and adherent to deep layers, with occasional discharge. MRI revealed a well-encapsulated lesion of 30 × 32 mm over metatarsals II to IV, with no tendon or bone involvement.

#### Surgical approach

**Figure 1:**
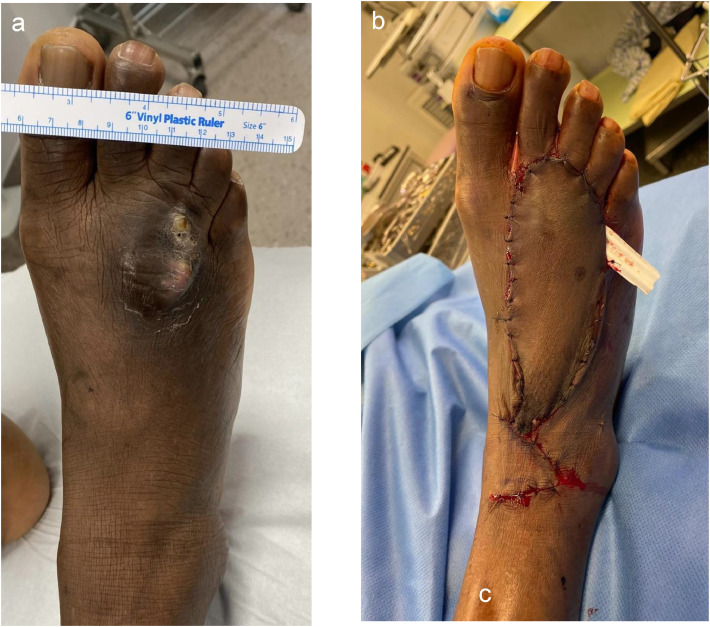
Pre, intra and Post operative presentation of Case 1. (a) Pre operative view with mycetoma; (b) Immediate postoperative appearance after reconstruction with SCIP free flap.

Wide excision with a 1 cm margin preserved the extensor tendons. A SCIP free flap from the left groin was chosen for its thinness and aesthetic match for dorsal foot reconstruction. The flap was anastomosed to the dorsal pedis artery and veins after opening the extensor retinaculum (Figure 1).

#### Postoperative course

No microsurgical complications occurred. After 5 days of bed rest and dangling protocol, rehabilitation began. The pathogen was Madurella Mycetomatis; antifungal therapy with itraconazole was initiated at 400 mg/day and maintained for 12 months. The patient was discharged on day 15. At 36 months, there were no signs of recurrence. Follow-up includes regular clinical evaluations and MRIs at 1 and 3 years.

### Case 2

#### Patient characteristics

A 47-year-old man from Ivory Coast, living in France since 2009, was referred in March 2022 for recurrent eumycetoma of the left foot. He had a BMI of 27.2 and a history of hypertension, corticosteroid-induced diabetes, and kidney transplantation in 2013. The onset date and traumatic event were unclear. He had prior surgery in 2020 and was treated with Voriconazole but was lost to follow-up.

#### Presentation and imaging

A painless 8 × 4 cm mass was noted, mobile on deep layers, with preserved joint function and sensation. MRI revealed recurrent multilocular lesions involving the extensor tendons (II–V) and osteitis of the fifth metatarsal. The previous pathogen identified was Phialemonium curvatum.

#### Surgical approach

**Figure 2:**
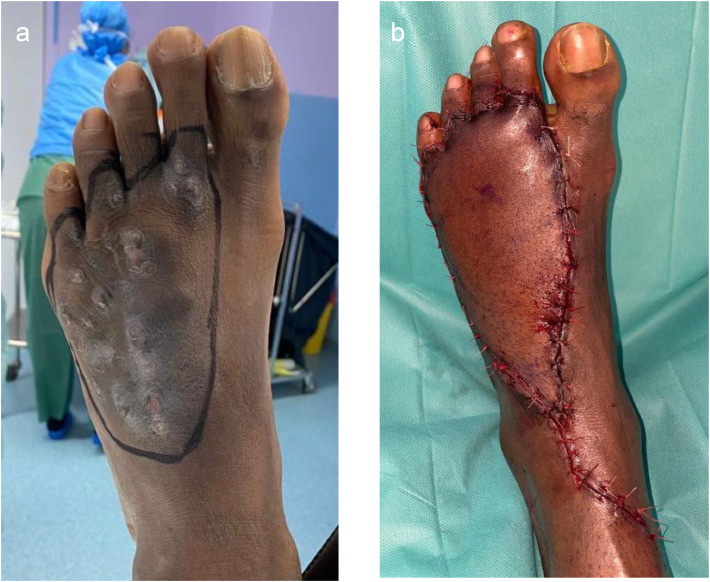
Pre, Intra and Post operative presentation of Case 2. (a) Pre operative drawing view with mycetoma; (b) Immediate postoperative appearance after reconstruction with ALT free flap.

A wide en-bloc excision with 1 cm safety margins included removal of the involved extensor tendons. Reconstruction was performed with a left anterolateral thigh (ALT) free flap due to its thickness and reliable pedicle. Anastomosis was completed with the dorsal pedis pedicle to cover the defect (Figure 2).

#### Postoperative course

Despite immunosuppression, the postoperative course was uneventful. After 5 days of strict decubitus and dangling protocol, rehabilitation started. The patient was discharged on day 8. Antifungal therapy with Isavuconazole was initiated at 200 mg/day and maintained for 12 months. One-year MRI showed no recurrence. At the 3-year follow-up, no clinical signs of disease were present.

## Discussion

Mycetoma is a neglected tropical disease, recently recognized by the WHO as a major health burden in endemic countries. Nevertheless, cases of eumycetoma are increasingly reported in nonendemic regions, including Europe, primarily due to migration. As global mobility increases, it is crucial that clinicians in nonendemic areas develop awareness of this condition, which is often misdiagnosed and mismanaged. Early diagnosis and adequate management can prevent extensive tissue destruction and reduce the risk of recurrence or mutilating surgeries.^1^^,^^4^^,^^6^

Management of eumycetoma is complex and requires a multidisciplinary approach. Although antifungal agents, particularly itraconazole and isavuconazole, have shown clinical efficacy, their ability to penetrate the characteristic fibrotic pseudocapsule of the lesion is limited. Thus, medical therapy alone is rarely curative. Radical surgical excision remains the cornerstone of treatment in most cases. However, achieving a balance between complete resection and preservation of limb function presents a significant challenge, especially in anatomically complex areas such as the foot.^3^^,^^8^

Historically, conservative or incomplete excision has been associated with high recurrence rates, approaching 50 % in some series. Both patients in our study had previously undergone surgical treatment with inadequate margins, leading to disease recurrence. These cases highlight the need for wide en-bloc excision with healthy tissue marginsideally including a layer of fascia as a natural barrier whenever feasible.^9^

One major limitation to radical surgery is the risk of large, complex soft tissue defects, which can threaten both function and aesthetics, and may ultimately necessitate amputation if coverage is not achievable. In this context, microsurgical reconstruction offers significant advantages. Free flaps allow for immediate closure of large defects, reduce healing time, and prevent secondary complications such as scar contraction, ulceration, or functional impairment. Furthermore, vascularized tissue may enhance thelocal immune response and facilitate delivery of systemic antifungals.^6^ Moreover, when the sole of the foot is affected, free flaps may be especially advantageous, since innervated flaps such as the anterolateral thigh or lateral arm (external brachial) flap can provide both durable coverage and restoration of protective sensation, which is essential for weight-bearing function.

Despite these potential benefits, microsurgical reconstruction in the context of mycetoma has been rarely reported. In the largest available series by Gismalla et al., which includes 34 patients, only one case was reconstructed with a free flap.^10^

In our experience, free flap reconstruction allowed for oncologic like resections with adequate margins, without compromising limb function or aesthetics. Flap selection was tailored to each case: a thin SCIP flap for superficial defects requiring contour preservatio, and a more robust ALT flap for deeper, voluminous lesions. Both procedures were well tolerated, even in an immunosuppressed patient, and no microsurgical complications were observed.

Our report supports the feasibility and safety of free flap reconstruction in the surgical management of eumycetoma in high-resource settings. However, in endemic and resource-limited settings, microsurgical reconstruction may not be feasible due to the lack of specialized expertise and infrastructure. In such contexts, alternative strategies such as local flaps, skin grafting, or staged procedures remain essential for limb preservation. Strengthening access to basic reconstructive techniques and antifungal therapy in endemic regions is therefore critical, and collaboration with referral centers may help bridge this gap.

Further studies with larger cohorts and longer follow-up are needed to assess long-term outcomes and recurrence rates in this population.

## Funding

None.

Informed consent was obtained from all individual participants involved in the study.

## Ethical approval

Not required.

## Declaration of competing interest

None.
